# Sensing of human stress biomarkers using BODIPY-functionalized carbon nanoparticles

**DOI:** 10.3389/fchem.2026.1849599

**Published:** 2026-08-25

**Authors:** Alessia Cavallaro, Michael Spinello, Victor Sebastian, Angelo Ferlazzo, Antonino Gulino, Roberta Ruffino, Giovanni Li Destri, Andrea Pappalardo, Nunzio Tuccitto, Giuseppe Trusso Sfrazzetto, Rossella Santonocito

**Affiliations:** 1 Department of Chemical Sciences, University of Catania, Catania, Italy; 2 Instituto de Nanociencia y Materiales de Aragoń Aragón (INMA), CSIC-Universidad de Zaragoza, Campus Rio Ebro, Zaragoza, Spain; 3 Department of Chemical and Environmental Engineering, Institute of Nanoscience and Materials of Aragon, Universidad de Zaragoza, Zaragoza, Spain; 4 Networking Research Center in Biomaterials, Bioengineering and Nanomedicine (CIBER-BBN), Instituto de Salud Carlos III, Madrid, Spain; 5 Laboratorio de Microscopías Avanzadas, University de Zaragoza, Zaragoza, Spain; 6 CSGI Consorzio Interuniversitario per Lo Sviluppo Dei Sistemi a Grande Interfase, Firenze, Italy

**Keywords:** BODIPY, carbon nanoparticles, fluorescence, nanosensor, stress biomarkers

## Abstract

Stress affects many aspects of human functioning, and its effects on cognition are particularly relevant in high performance scenarios such as military operations and space missions. Dopamine, noradrenaline, and cortisol are key biomarkers associated with stress, anxiety, depression, and cardiovascular disease, yet their qualitative and quantitative detection usually requires lengthy laboratory analyses and lacks a single, selective one-pot method. In this work, we developed a fluorescent nanosensor based on carbon nanoparticles functionalized with a BODIPY-based fluorophore (CNPs-Ar-BDPy) for the detection and discrimination of dopamine, noradrenaline, and cortisol in aqueous solution. The sensing platform exploits the carbon nanoparticle core as a structural scaffold and the BODIPY moiety as a recognition site, enabling specific interactions with catecholamines and cortisol through hydrogen bonding between the BODIPY fluorine atoms and the OH and C=O groups of the analytes. The CNPs-Ar-BDPy were synthesized, functionalized, and fully characterized by UV Vis and fluorescence spectroscopy, and their sensing performance was evaluated in aqueous media using fluorescent titration and FT-IR. Selectivity was further assessed using multivariate analysis by PLS-DA, demonstrating effective discrimination among the three biomarkers. In addition, the role of the covalent linkage between the carbon nanoparticle core and the BODIPY unit was investigated, and the obtained apparent affinity constants were compared with those of analogous nanoprobes featuring a non-rigid, non-preorganized spacer. The rigid aryl linked BODIPY proved to enhance binding by about two orders of magnitude, highlighting the importance of structural preorganization and fluorescence continuity across the core fluorophore interface. The proposed nanosensor thus represents a promising tool for rapid, selective monitoring of stress related biomarkers.

## 1 Introduction

Catecholamine neurotransmitters such as dopamine (DA) and norepinephrine (NAdr) are key regulators of cardiovascular, metabolic, endocrine, and central nervous system functions, and their dysregulation is associated with a broad spectrum of pathologies, including Parkinson’s disease, Schizophrenia, depression, hypertension, pheochromocytoma, and other neurodegenerative or neuroendocrine disorders (Martin et al., 1984; Tong et al., 2019; Wackerhage et al., 2022).

Cortisol is the key stress hormone involved in circadian rhythm, immune modulation, and metabolic regulation. Because alterations in catecholamine and cortisol levels in biological fluids (Supplementary Table S1 in the Supplementary Material) can provide early warning of disease onset and progression, their reliable quantification has become an important goal in both clinical diagnostics and translational research (Drujan, et al., 1959; Pluto and Bürger, 1988; Schwab et al., 1992). Conventional analytical techniques for catecholamine and cortisol determination, such as High-Performance Liquid Chromatography (HPLC) (HPLC) (Was et al., 2019), liquid chromatography–mass spectrometry (LC–MS), and electrochemical methods, generally offer excellent sensitivity and selectivity but require sophisticated instrumentation, trained personnel, time-consuming sample pretreatment, and are not easily implemented as portable or *point-of-care* (PoC) tools (Drujan et al., 1959; Pluto and Bürger, 1988; Schwab et al., 1992; Was et al., 2019; Wei et al., 2005; Santonocito et al., 2024a). Electrochemical sensors based on metal or metal-oxide nanoparticles, Carbon nanoparticle conjugates (CNPs) with DNA aptamers (Khramtsov et al., 2018; Wang et al., 2011) and carbon nanostructures can simplify instrumentation, but they often suffer from matrix effects, or limited ability to discriminate structurally similar catecholamines (Puglisi et al., 2024a) without careful surface engineering and potential interference from common coexisting species such as ascorbic and uric acids. High-Performance liquid chromatography requires extensive sample pretreatment and is not easily implemented in portable or point-of-care formats. In contrast, metaloxide nanoparticles and carbon nanostructures can simplify instrumentation, but they often suffer from matrix effects or a limited ability to discriminate structurally similar analytes (Tran et al., 2026).

In this context, optical based nanoparticles have emerged as attractive alternatives owing to their high sensitivity (Chandra, et al., 2011; Darwish, et al., 2024), rapid response, and compatibility with miniaturized or smartphone assisted readout platforms (Tran et al., 2026, Santonocito et al., 2024b). Among these, carbon nanostructures (Li et al., 2025a), especially carbon nanoparticles (CNPs) and related carbon based nanozymes, have drawn particular attention due to their favorable water solubility, low toxicity, excellent photostability, tunable surface chemistry, and the possibility to tailor emission properties and catalytic activity through heteroatom doping or surface functionalization (Ayanda, et al., 2024; Bao et al., 2025). They have significant, practical and economic applications in the fields of biosensing, optical imaging and medical diagnostics (Cavallaro et al., 2026).

Nitrogen- and phosphorus-codoped carbon dots, for example, can reach quantum yields above 50%–80% and have been successfully exploited as fluorescent probes or nanozyme mimics for off–on sensing of dopamine and other catecholamine neurotransmitters via Fe^3+^-mediated quenching–recovery or Fenton-like mechanisms (Shi et al., 2016; Guo et al., 2015; Chen et al., 2026; Luo et al., 2025; Li et al., 2025b).

Recent studies have further demonstrated that CNPs-based or broader carbon nanostructure-based platforms (Agrawal and Sethy, 2025; Asadian et al., 2019) can be engineered into single well or paper-based arrays capable of discriminating against multiple catecholamines through multichannel colorimetric or ratiometric fluorescence readouts, often combined with chemometric analysis and smartphone imaging for onsite quantification (Zhang et al., 2019; Qiu et al., 2012; Puglisi et al., 2024b). Recent optical platforms exploit CNPs combined with cortisol selective aptamers to achieve “turn–on” or “turn–off” fluorescence responses, typically via inner filter or FRET type quenching in the CNP-quencher complex that is reversed upon specific binding of cortisol (Hameed et al., 2021; Shi et al., 2015; Yang et al., 2023). However, many of these systems rely on relatively complex multicomponent formulations, require elevated temperatures or long reaction times, or are optimized for a single catecholamine or cortisol in buffered solutions rather than simultaneous, quantitative analysis of several catecholamines in complex biological matrices. CNPs-based (-nanostructure-based -well or paper-based) arrays also exist that can discriminate multiple catecholamines through multichannel colorimetric or ratiometric fluorescence readouts (Bao et al., 2025; Hassani-Marand et al., 2023), often combined with chemometric analysis and smartphone imaging for on-site “turn-on” or “turn-off” fluorescence responses, typically via inner filter or FRET-type quenching in the CNPs.

Thus, the possibility to detect these stress biomarkers, with one single analysis, in a fast and easy methodology is of primary interest and importance. Fluorescent nanoparticle sensors that combine high quantum yield (Baker and Baker, 2010; Trusso Sfrazzetto and Santonocito, 2022), robust colloidal and photochemical stability, and well-defined, analyte-dependent emission changes therefore remain highly desirable for advancing multiplexed, minimally invasive monitoring of catecholamines and cortisol. Building on these advances, this paper describes the design, synthesis and characterization of a sensor based on fluorescent nanoparticles for the selective detection of catecholamine neurotransmitters and cortisol, with a particular focus on exploiting the surface chemistry of nanoparticles and supramolecular interactions with bioanalytical applications and future *point-of-care* sensor applications.

In particular, we present a new optical nanosensor for the detection of DA, NAdr, and cortisol. The nanosensor is based on carbon nanoparticles functionalized on their outer shell with a BODIPY-based fluorophore (*CNPs-Ar-BDPy*) capable of acting as a recognition site. After the synthesis, functionalization, morphological and structural characterization of *CNPs-Ar-BDPy*, their use as DA, NAdr and cortisol sensors was tested in aqueous solution. Selectivity was also evaluated through fluorescence studies by multivariate analysis (PLS-DA). FT-IR studies were conducted to investigate the detection mechanism, supporting the hypothesis of hydrogen bond formation between the catechol portion of dopamine and the fluorine atoms of *CNPs-Ar-BDPy.*

We studied the effect of the covalent linkage between carbon nanoparticle core and Bodipy moiety to better understand the mechanism of the recognition and sensing event. Finally, we studied the sensor’s capabilities by comparing the apparent affinity constants obtained with the apparent affinity constants of other fluorescent CNPs with a non-rigid and non-preorganized arm, demonstrating the importance of having fluorescence continuity between the core of the nanoparticles and the BDPy, which leads to a two-order-of-magnitude increase in apparent affinity constants.

## 2 Materials and methods

### 2.1 General experimental methods

All chemicals were reagent-grade, acquired by Sigma-Aldrich and used without further purification. For the synthesis of aryl-substituted BODIPY alcohol (BDPy-Ar-OH) and BDPy-CH_2_-Cl_2_, the experimental procedures reported in the literature were followed (Santonocito et al., 2025).

The ^1^H-NMR experiments were carried out at 27 °C on a Varian UNITY Inova 500 MHz spectrometer (International Equipment Trading Ltd., Mundelein, IL, USA) at 499.88 MHz equipped with a pulse field gradient module (Z axis) and a tunable 5 mm Varian inverse detection probe (ID-PFG).

UV-Vis measurements were taken using a JASCO V-750 UV-vis double-beam spectrophotometer equipped with a 1 cm path-length cell (resolution 0.1 nm), luminescence measurements were performed on a JASCO FP-8550 spectrofluorometer (resolution 0.5 nm) at room temperature. Emission signals were collected at a 90° angle with respect to the excitation path, using slit widths of 2.5 nm for both excitation and emission.

FT-IR spectra were acquired by using a PerkinElmer Spectrum One spectrophotometer. For the analysis of the CNPs, samples of CNPs-5FPh and *CNPs-Ar-BDPy* were prepared as KBr pellets, using KBr as the solid dispersant medium (ratio sample/KBr 1:100). Measurements were acquired in transmission over the range 4,000-450 cm^-1^, with an average of 16 scans per sample.

X-ray photoelectron spectra (XPS) were measured at a 45° take-off angle relative to the surface sample holder, with a PHI 5000 Versa Probe II system (ULVAC-PHI, INC., base pressure of the main chamber 1 × 10^−8^ Pa) (Briggs and Grant, 2004; Gulino, 2013). FT-IR measurements were performed using a Perkin Elmer Spectrum One Spectrophotometer.

Morphological characterization was carried out in tapping mode using a Nanoscope IIA-MultiMode Atomic Force Microscope (AFM), Digital Instruments (Santa Barbara, CA, USA). Images were acquired at a scan rate of 1 Hz, with a resolution of 512 × 512 pixels per image using Tap 300 G silicon probes (Budget sensors) mounted on cantilevers with a nominal force constant of 40 Nm^-1^ and a resonant frequency of 300 kHz.

### 2.2 Synthesis of CNPs-COOH

Powder citric acid (30 g, 0.15 mol) was placed in a beaker and heated on a heating plate at 300 °C until caramelization occurred. Once room temperature was reached, a 0.25 M NaOH aqueous solution (200 mL) was slowly added under magnetic stirring, obtaining a solution that was centrifuged at 3,000 rpm for 30 min. The supernatant was transferred into a dialysis membrane (cut-off 12–14 kDa). After 2 days of dialysis, the content of the membranes was dried under vacuum, yielding CNPs-COOH. FTIR characterization of CNPs-COOH was according to literature data (Cavallaro et al., 2026).

### 2.3 Synthesis of CNPs-5FPh

700 mg of CNPs-COOH were dissolved in 5 mL of pentafluorophenol (5FPh) by heating at 50 °C under magnetic stirring. After complete solubilization, 817 mg (4.3 mmol) of 1-Ethyl-3-(3dimethylaminopropyl) carbodiimide hydrochloride (EDC x HCl) were added under N_2_ atmosphere. After 48 h, the reaction mixture was cooled to room temperature and then solubilized with 25 mL of dichloromethane. Then, a liquid-liquid extraction with basic water (pH∼9) was performed (three times). The organic phase was dried under vacuum, obtaining 760 mg of CNPs-5FPh.

### 2.4 Synthesis of *CNPs-Ar-BDPy*

CNPs-5FPh (760 mg) were solubilized with 5 mL of dry CH_2_Cl_2_. Then, 380 mg of BDPy-Ar-OH (5 eq, 0.95 mmol) and 800 µL of DIPEA (4.8 mmol) were added to the solution. The reaction mixture was stirred at room temperature under N_2_ atmosphere for 3 days. The solvent was removed under vacuum, and the solid was solubilized in water. Then, a liquid-liquid extraction with basic water (pH∼8) was performed (three times). The organic phase was dried under vacuum, The solution was transferred into a membrane (cut-off 12-14 KDa), and dialysis was carried out for 3 days to purify the nanoparticles. Characterization of *CNPs-Ar-BDPy* was performed using AFM, TEM, ^1^H NMR, XPS, FT-IR, UV-vis and fluorescence. ^1^H NMR (500 MHz, CDCl_3_): δ 0.98 (t, *J* = 7.5 Hz, 6H), 1.35 (s, 6H), 2.30 (q, *J* = 7.5 Hz, 4H), 2.52 (s, 6H), 6.94 (d, *J* = 8.8 Hz, 2H), 7.06 (d, *J* = 8.8 Hz, 2H) ppm (details in Supplementary Figure S1).

### 2.5 Synthesis of CNPs-CONH(CH_2_)_2_OH

290 mg of CNPs-COOH were dissolved in 20 mL of ethanolamine by heating at 130 °C under magnetic stirring. After 48 h, the reaction mixture was cooled to room temperature (details in Supplementary Scheme S1a). Characterization of CNPs-CONH(CH_2_)_2_OH was performed using ^1^H NMR and is according to the literature (Tuccitto et al., 2020). ^1^H NMR (500 MHz; D_2_O):δ 3.13 (t, J = 4.89 Hz, 2H), 3.82 (t, J = 4.89 Hz, 2H) details in Supplementary Figure S21.

### 2.6 Synthesis of *CNPs-Ethyl-BDPy*

CNPs-CONH(CH_2_)_2_OH (160 mg) were solubilized with 5 mL of dry DMSO. Then, 50 mg of BDPyCH_2_-Cl_2_ (0.14 mmol) and 800 µL of DIPEA (4.8 mmol) were added to the solution (details in Supplementary Scheme S1b). The reaction mixture was stirred at room temperature under N_2_ atmosphere for 3 days. The solvent was removed under vacuum, and the solid was dispersed in water. Then, a liquid-liquid extraction with dichloromethane was performed (three times). The organic phase was dried; the solvent was removed under vacuum. The crude product was transferred into a membrane (cut-off 12-14 KDa), and dialysis was carried out in ethanol for 3 days to purify the nanoparticles. Characterization of *CNPs-Ethyl-BDPy* was performed using ^1^H NMR, UV-Vis and fluorescence. ^1^H NMR (500 MHz; CDCl_3_):δ 1.05 (t, J = 7.9 Hz, 6H); 2.39 (s, 6H); 2.41 (q, J = 7.9 Hz, 4H); 2.50 (s, 6H); 2.92 (t, J = 4.9 Hz, 2H); 3.73 (t, J = 4.9 Hz, 2H); 4.01 (s, 2H) ppm, details in Supplementary Figure S22.

### 2.7 XPS characterization

X-ray photoelectron spectra (XPS) were measured at a 45° take-off angle relative to the surface sample holder, with a PHI 5600 Multi Technique System which offers a good control of the photoelectron take-off angle (base pressure of the main chamber 2 × 10^−10^ Torr). The acceptance angle of the analyzer and the precision of the sample holder concerning the angle are ± 3° and ± 1°, respectively. The spectrometer is equipped with a dual anode X-ray source; a spherical capacitor analyzer (SCA) with a mean diameter of 279.4 mm; an electrostatic lens system Omni Focus III. Samples were deposited on Si (100) substrates and mounted on Mo stubs. Spectra were excited with Al-Kα X-ray radiation using a pass energy of 5.85 eV. Structures due to Kα_2_ satellite radiation were subtracted from the spectra prior to data processing. No relevant charging effect has been observed. The instrumental energy resolution was ≤ 0.5 eV. The XPS peak intensities were obtained after Shirley’s background removal (Briggs and Grant, 2004; Gulino, 2013, Gulino and Fragalà, 2005). Spectra calibration was achieved by fixing the Ag 3d_5/2_ peak of a clean sample at 368.3 eV (Greczynski and Hultman, 2020). The atomic concentration analysis was performed by considering the relevant atomic sensitivity factors. The fitting of some XP spectra was carried out, using the XPSPEAK4.1 software, by fitting the spectral profiles with Gaussian envelopes, after subtraction of the background. This process involves data refinement, based on the method of the least squares fitting, carried out until there is the highest possible correlation between the experimental spectrum and the theoretical profile. The residual or agreement factor *R*, defined by *R* = [Σ (F_obs_ − F_calc_)^2^/Σ (F_obs_)^2^]^1/2^, after minimization of the function Σ (F_obs_ − F_calc_)^2^, converged to the value of 0.03.

### 2.8 FT-IR measurements

Solutions of *CNPs-Ar-BDPy* and DA (1 mg/mL and 1.0 × 10^−3^ M respectively) were prepared in anhydrous CH_2_Cl_2_. For each compound, 50 µL of the solutions were laid on a 2 × 1 cm silicon wafer. After evaporation of the solvent at air, a thin layer of the compound remained on the silicon wafer; FTIR measurements were taken, thus giving a spectrum of each compound. Then, nanosensor/analyte complexes were formed, mixing their solutions in 1:1 stoichiometry. 50 μL of each complex were laid on the silicon wafer, and the solvent was removed in the air. Then, spectra of *CNPs-Ar-BDPy*@DA was recorded (details in Supplementary Figure S5).

### 2.9 Morphological characterization (AFM and TEM)

AFM samples were prepared by rapidly drop-casting a few microliters of a 1:10 pre-diluted aqueous dispersion of *CNPs-Ar-BDPy* onto freshly cleaved mica substrates.

The CNPs were examined by transmission electron microscopy (TEM) using a Tecnai T20 operated at 200 kV and a 300 kV field-emission gun (FEG) TEM equipped with a SuperTwin® lens, providing a point resolution of 1.9 Å and a lattice resolution of 0.19 nm. TEM grids were prepared by depositing 25 µL of the CNPs suspension onto a carbon-film-coated 200-mesh copper grid, followed by drying at room temperature for 4 h.

### 2.10 UV-visible and fluorescence measurements

The UV–Vis absorption spectra of *CNPs-Ar-BDPy* and *CNPs-Ethyl-BDPy* (see Supplementary Figures S7, 23 respectively) were performed in quartz cuvettes using MilliQ water solution of *CNPs-ArBDPy* (0.1 mg/mL) and ethanol solution of *CNPs-Ethyl-BDPy* (1 mg/mL), recording spectra over the 200–700 nm range.

Fluorescence measurements were carried out at room temperature with excitation and emission slit widths set to 2.5 nm. A *CNPs-Ar-BDPy* stock solution (1 mg mL^-1^ in MilliQ water, pH 7) was diluted 10-fold to obtain the 0.1 mg mL^-1^ working concentration used for fluorescence experiments and titrations. For emission screening, the excitation wavelength (λ_ex_) was scanned from 280 to 460 nm. Titrations with DA, NAdr, and cortisol were conducted at a fixed λ_ex_ of 370 nm by adding aliquots (2–100 µL) of a 10^-4^ M solutions in MilliQ water directly into the cuvette. Fluorescence spectra were acquired in triplicate over an analyte concentration range (dopamine, norepinephrine, and cortisol) of 0.01 µM–5 µM. For titrations between BDPy-Ar-OH and DA, NAdr and Cortisol, we used an ethanol solution [BDPy-Ar-OH] = 1 × 10^−6^ M, using an λ_ex_ of 500 nm, adding aliquots (2–100 µL) of a 10^-4^ M solutions of DA, NAdr and cortisol in ethanol directly into the cuvette.

Apparent affinity constants (log β) were obtained using HypSpec (v1.1.33) (Legnani et al., 2020, Puglisi et al., 2019b, Puglisi et al., 2019a) by fitting solution spectrophotometric data to an assumed complexation model through a least-squares refinement, which yields both the component spectra and the corresponding stability constants. Fit quality was evaluated using a chi-square (χ^2^) test assessing whether residuals follow a normal distribution, in this work, χ^2^ values were below 10 in all cases, based on three independent measurement sets. For the apparent affinity constants calculations, the molar concentration of *CNPs-Ar-BDPy* provided to the software was set to 10^-7^ M, as estimated from assumptions and calculations reported in the literature (Cavallaro et al., 2026). The affinity constants reported here should be regarded as apparent constants, since the concentration of the nanosensor was estimated according to literature procedures rather than directly measured.

The detection limit (LOD) was calculated using the formula LOD = 3σ/k were where: 3 represents the IUPAC safety factor that allows the presence of an analyte to be identified with a confidence level of 99.7%; σ is the standard deviation of 10 independent measurements of the background (blank) signal; and k is the sensitivity, i.e., the slope of the line (emission intensity at λ max vs. added equivalents).

## 3 Results and discussion

### 3.1 Design and synthesis of CNPs-Ar-BDPy

From a chemical point of view, catecholamines and cortisol are characterized by the presence of different hydroxyl groups, phenolic (in the catecholamines) and aliphatic (in cortisol). Thus, these biomarkers can be detected exploiting non-covalent interactions with these functional groups. In particular, hydrogen bond is particularly useful to this end due to the stability and directionality, also in the water environment if incorporated into a nanoparticle sensor (Puglisi et al., 2024a; Tuccitto et al., 2020) In this design, the carbon nanoparticle core plays a dual role: it contributes intrinsic fluorescence through its characteristic emission profile and serves as a versatile scaffold for multivalent sensing. The BODIPY-OH fluorophore (BDPy-Ar-OH) was chosen as the recognition element because it can establish hydrogen-bond interactions between its fluorine atoms and the phenolic groups of catecholamines, as well as the hydroxyl groups of cortisol (Puglisi et al., 2024b). By anchoring multiple recognition units onto a single nanostructure, this approach exploits nanoscale proximity effects and thereby enhances selectivity toward catecholamines and cortisol (Santonocito et al., 2022).

**SCHEME 1:**
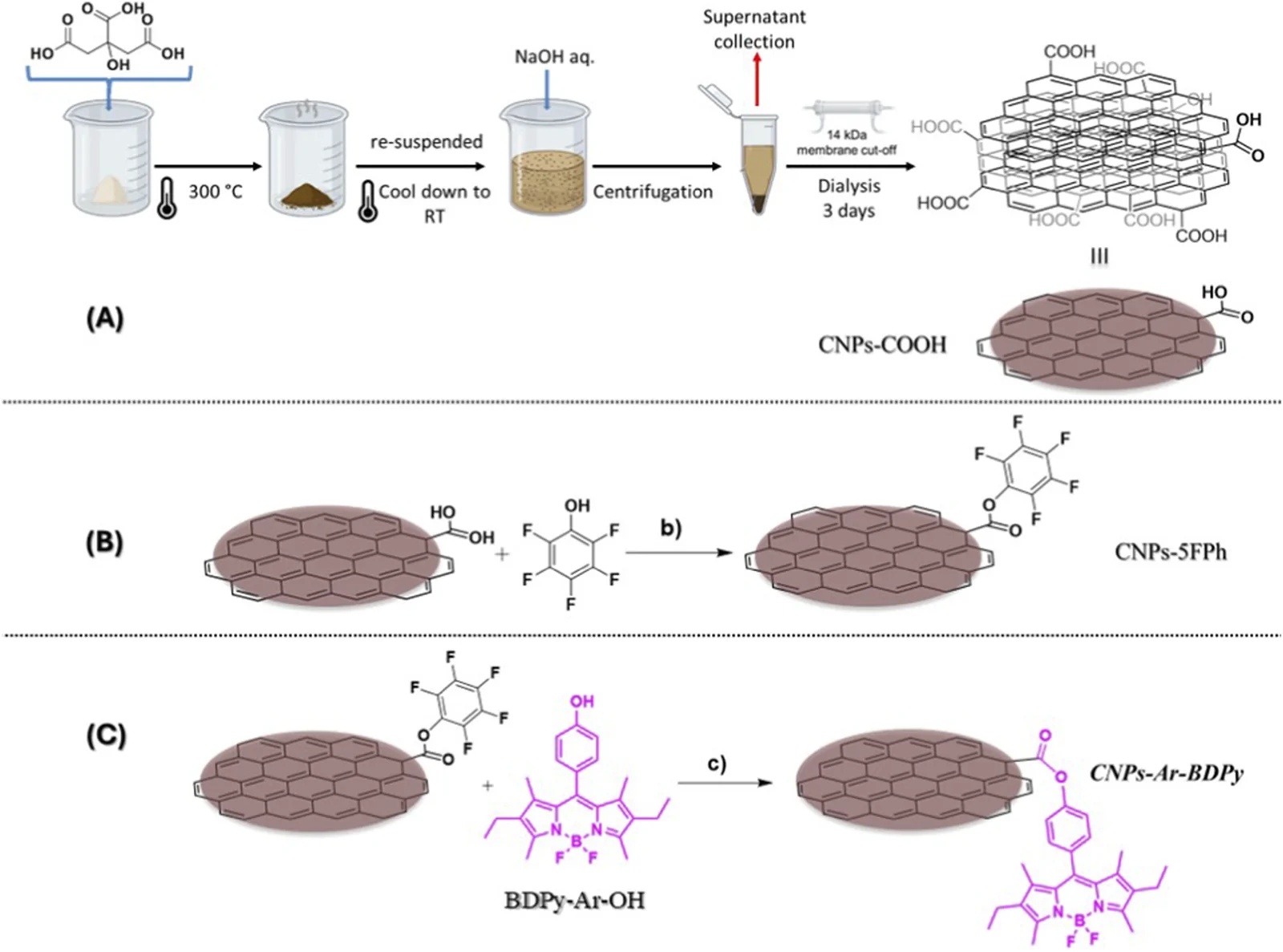
Schematic pathway showing the reactions to obtain *CNPs-Ar-BDPy*: **(A)** hydrothermal synthesis and purifications steps of CNPs-COOH **(B)** EDC, N_2_, RT. **(C)** DIPEA, CH_2_Cl_2_ dry, RT, N_2_, 3 days.

*CNPs-Ar-BDPy* were synthesized in three steps (Scheme 1). First, carboxylate CNPs (CNPs-COOH) were obtained by solvent-free carbonization of citric acid at 300 °C, affording a dark-brown residue that was subsequently dissolved in aqueous NaOH (Scheme 1A). The crude material was purified by centrifugation to remove larger particulates and by dialysis to eliminate low-molecular-weight species, providing CNPs with a uniform morphology. Owing to the citric-acid-derived framework, the resulting nanoparticles display a high density of surface carboxyl groups that serve as reactive handles for further functionalization (Li-Destri et al., 2019).

In the second step, the surface carboxyl groups were activated by reaction with pentafluorophenol (5FPh), which act also as solvent, in the presence of a carbodiimide coupling reagent (Scheme 1B). The resulting CNPs-5FPh intermediate is soluble in CH_2_Cl_2_, which enables subsequent nucleophilic substitution with the recognition unit. Finally, CNPs-5FPh were reacted with the BDPy-Ar-OH derivative, using DIPEA to enhance the nucleophilicity of the amine functionality (Scheme 1C). The aryl linkage is introduced through the BDPy-Ar-OH derivative used in this coupling step. The crude product was dialyzed against water for 3 days to afford purified *CNPs-Ar-BDPy*.

### 3.2 Characterization of *CNPs-Ar-BDPy*

Once synthesized, *CNPs-Ar-BDPy* were characterized to investigate chemical structure, morphology and optical behavior.

#### 3.2.1 Chemical characterization (^1^H NMR, XPS and FT-IR)

The ^1^H NMR spectrum of *CNPs-Ar-BDPy* (recorded in CDCl_3_) shows the set of resonances attributable to the BDPy-Ar-OH fluorophore, and these signals are shifted towards low ppm compared to those of free BDPy (Supplementary Figure S1). This is consistent with the conversion of the phenolic -OH of BODIPY to an ester, supporting the successful functionalization of the nanoparticle surface with BDPy.

**FIGURE 1:**
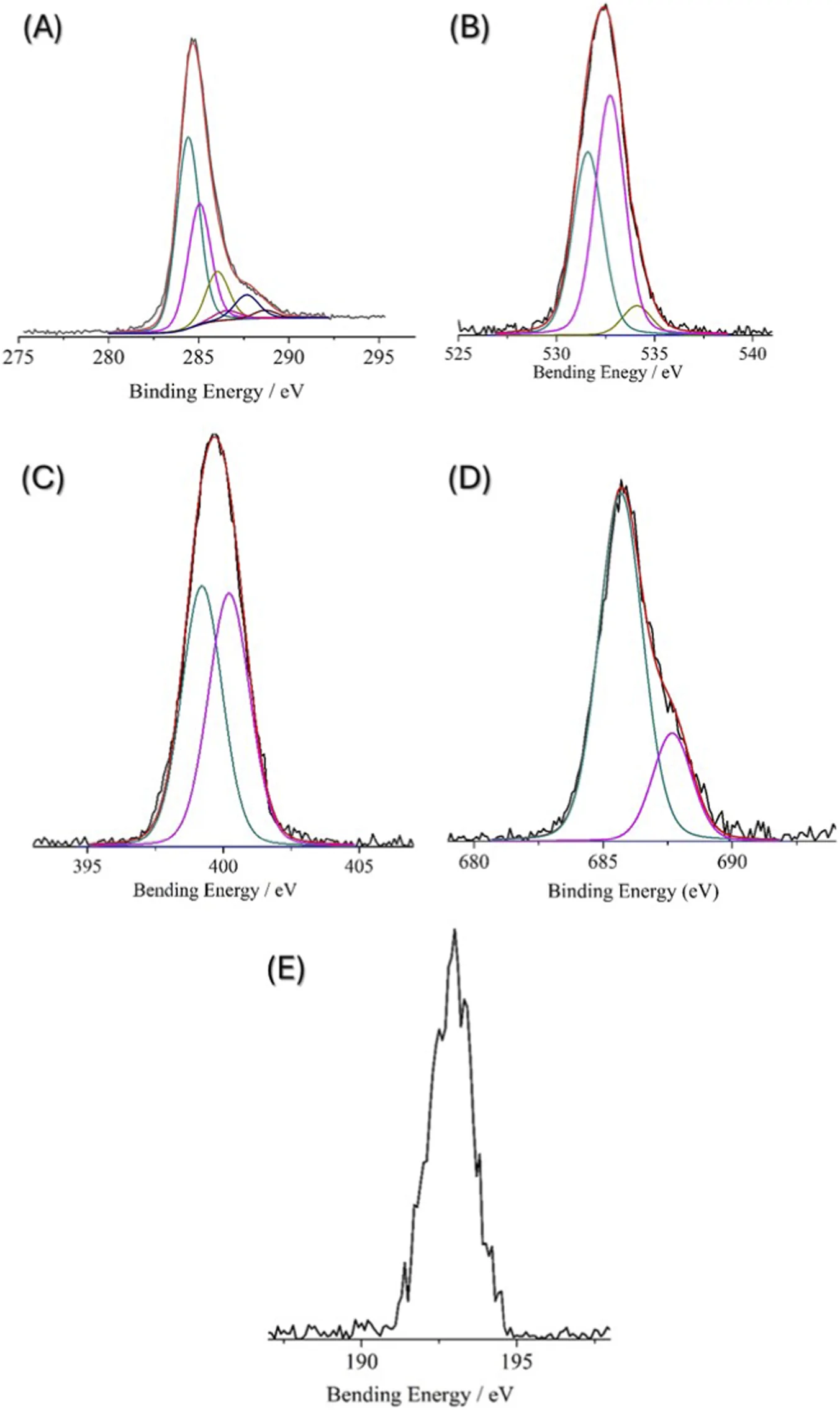
**(A)** Al Kα excited XPS of *CNPs-Ar-BDPy* sample in the C 1s binding energy region: The dark cyan, magenta, dark yellow, wine, pink and navy lines refer to the 284.7, 285.0, 286.0, 286.9, 288.2, and 289.1 eV Gaussians components, respectively. **(B)** Al Kα excited XPS of *CNPs-Ar-BDPy* sample in the O 1s binding energy region: The dark cyan, magenta, and dark yellow lines refer to the 531.6, 532.7, 534.1 eV Gaussians components, respectively. **(C)** Al Kα excited XPS of the *CNPs-ArBDPy* sample in the N 1s binding energy region: The dark cyan, and magenta lines refer to the 399.3, and 400.2 eV Gaussians components, respectively. **(D)** Al-Kα excited XPS of *CNPs-Ar-BDPy* sample in the F 1s binding energy region. The dark cyan, and magenta lines refer to the 685.9, and 687.7 eV Gaussians components, respectively. **(E)** Al-Kα excited XPS of *CNPs-Ar-BDPy* sample in the B 1s binding energy region. The blue line represents the background, and the red line superimposed to the experimental black profile refers to the sum of the Gaussian components.

Figure 1A shows the XP spectrum of *CNPs-Ar-BDPy* in the C 1s binding energy region. A careful deconvolution of the experimental spectrum required five Gaussians at: 284.7 eV due to the C sp2 states of the CNPs structure (relative intensity 44%), 285.0 eV due to the C-C and C-H sp3 states (relative intensity 29%), 286.0 eV due to the C-N states of the pyrrole ring in the BDPy functional group (relative intensity 12%), 286.9 eV due to the C-O states of the C of the BDPy ester group (relative intensity 3%), 288.1 eV due to the -COOH states of the carboxyl groups (relative intensity 12%) (Schiavo et al., 2025) and 289 (Briggs and Grant, 2004), due to aromatic carbon of some remaining pentafluoro phenol reagent (relative intensity 2.5%) (Buschbeck et al., 2019; Gulino et al., 2005). Figure 1B shows the XP spectrum of *CNPs-Ar-BDPy* in the O 1s binding energy region. The spectral fitting required three Gaussians at: 531.6 eV, due to the -C=O states of the carboxyl group of the BDPy (relative intensity 40.7%), and 532.7 eV attributable to the -C-O-BDPy group (relative intensity 53.1%), and 534.1 eV due to phenolic oxygen of some remaining pentafluoro phenol reagent (relative intensity 6.2%) (Lapuente, et al., 2003). Figure 1C shows the XP spectrum of *CNPs-Ar-BDPy* in the N 1s binding energy region. The accurate spectral fitting required two Gaussians at: 399.3 eV due to the neutral nitrogen present in the pyrrole ring of BDPy, and 400.2 eV due to the formally quaternized pyrrole. A comparison of the relative intensity areas shows a ratio of 1:1 (Tikhonov et al., 2023). Figure 1D shows the XP spectrum of *CNPs-Ar-BDPy* sample in the F 1s binding energy region. The accurate spectral fitting required two Gaussians at: 685.9 eV, attributable to fluorine of the bodipy bound to the carbon nanoparticles (Gulino et al., 2005; Perricelli et al., 2023) and 687.7 eV due to some residual pentafluorophenol reactant used during the *CNPs-Ar-BDPy* synthesis. Figure 1E shows the XP spectrum of *CNPs-Ar-BDPy* sample in the B 1s binding energy region, which consists of a single peak at 193.0 eV, attributable to the boron atom of BDPy (Tikhonov et al., 2023).

The XPS atomic concentration analysis of *CNPs-Ar-BDPy* revealed a B/F ratio of 0.4 (nominal expected ratio = 0.5) due to some remaining pentafluorophenol reactant in the sample, while the B/O ratio was found = 0.11. Considering that there are two oxygen atoms per -COOH group, it emerges that the 22% of the -COO groups present on the surface of the CNPs have been functionalized with the BPDy. The B/C ratio was 0.028. Considering that, the BDPy itself contains 23 carbon atoms per molecule (8 sp3 and 15 sp2 C atoms), a tentative calculation of the functionalization of the carbon atoms of the CNPs surface with -COO groups give about a 12% value. Since XPS is a technique that probes just a surface thickness of a few nanometers and, consequently, the above reported values refer to the topmost *CNPs-Ar-BDPy* surface.

FT-IR spectroscopy was used to confirm successful surface functionalization. In particular, comparison of the spectra of CNPs-5FPh and *CNPs-Ar-BDPy* (Supplementary Figures S3-S5) shows that the bands at 996 and 1,009 cm^-1^, assigned to 5FPh C–F stretching modes, disappear, which is consistent with consumption of the 5FPh group and thus with occurrence of the substitution reaction. Additionally, the formation of the ester bond between CNPs and BDPy is supported by the appearance of a band at 1,192 cm^-1^, attributable to the asymmetric stretching of C–C (=O)–O–C in esters, furthermore, there is a band at 1,279 cm^-1^ attributed to B-F stretching and another at 840 cm^-1^ attributed to B–F vibration (Ryabchenko et al., 2017).

#### 3.2.2 Morphological characterization (AFM and TEM)

**FIGURE 2:**
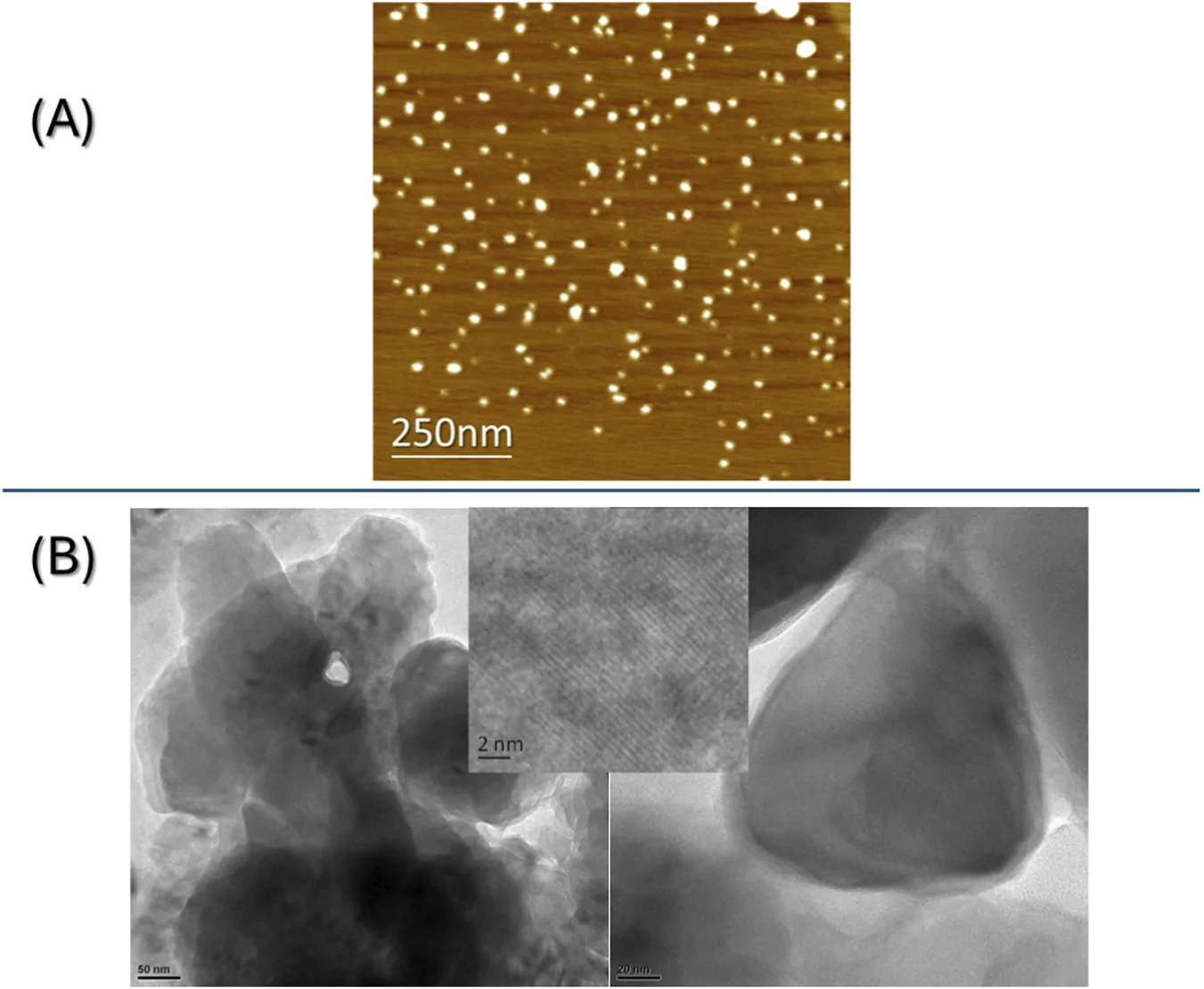
Representative AFM **(A)** and TEM **(B)** images of *CNPs-Ar-BDPy*.

The size and morphology of *CNPs-Ar-BDPy* were first explored by means of AFM imaging (Figure 2A; Supplementary Figure S6) performed on a dry sample, indicating the presence of several flat irregularly shaped particles. The AFM analyses performed on the original *CNPs-Ar-BDPy* show an average high of 2.75 ± 0.52 nm and a diameter of 22.27 ± 3.68 nm.

The images in Figure 2B show representative TEM micrographs of *CNPs-Ar-BDPy*, revealing two main morphological populations. Transmission electron microscopy images of the *CNPs-Ar-BDPy* reveal a tendency toward aggregation, which is likely induced during sample preparation, particularly upon solvent evaporation on the TEM grid. Despite this aggregation, individual nanostructures can still be discerned, exhibiting a polymorphic carbon framework composed of graphitic domains distributed within amorphous regions. High-resolution TEM analysis further reveals the presence of lattice fringes with an average interplanar spacing of ∼0.34 nm, which can be assigned to the (002) basal planes of graphitic carbon (Cavallaro et al., 2026). These ordered domains coexist with less structured regions, including areas of turbostratic carbon, consistent with the partial structural ordering typically observed in carbon materials derived from the pyrolysis of citric acid. Importantly, no distinct phase segregation or organic deposits attributable to BDPy are detected, suggesting a homogeneous surface functionalization rather than the formation of separate molecular aggregates.

#### 3.2.3 Optical characterization (UV-Visible and fluorescence spectroscopy)

The optical properties of *CNPs-Ar-BDPy* were evaluated using UV-Vis and fluorescence spectroscopy. The absorption spectrum was recorded using a concentration of 0.1 mg mL^-1^ in MilliQ water at pH = 7; a broad absorption profile from 220 to 480 nm can be observed, which can be attributed to the π-π* and n-π* electronic transitions of the conjugated C=C domains (Supplementary Figure S7a) typical of graphitic carbon nanoparticles (Cavallaro et al., 2026; Tuccitto et al., 2020). In addition, the UV-Vis spectrum shows an intense band centered at 540 nm, typical of the BDPy fluorophore (Lim et al., 2026). The fluorescence spectra of *CNPs-Ar-BDPy* were recorded using a concentration of 0.01 mg mL^-1^ in MilliQ water (Supplementary Figure S7b), in the λ_ex_ range of 280–460 nm. The emission spectrum of *CNPs-Ar-BDPy* remains constant, so it can be assumed that the nanoparticles are stable over this range of time, 7 days (see Supplementary Figure S28).

The emission maximum centered at 425 nm, attributable to the CNPs core, decreases with increasing excitation wavelength, while the BDPy maximum at 540 nm increases with increasing λ_ex_. The maximum emission centered at 425 nm does not vary with λ_ex_. This behavior may be attributed to the reduced polydispersity resulting from the dialysis process, as also confirmed by TEM analyses, which likely decreases the number of emissive states (Ullal, et al., 2024). Together with the relatively large size of these CNPs, this may have contributed to the excitation-independent emission. The narrow band that systematically follows the excitation wavelength is characteristic of Raman scattering water and is regularly observed in fluorescence experiments.

### 3.3 Sensing mechanism

**FIGURE 3:**
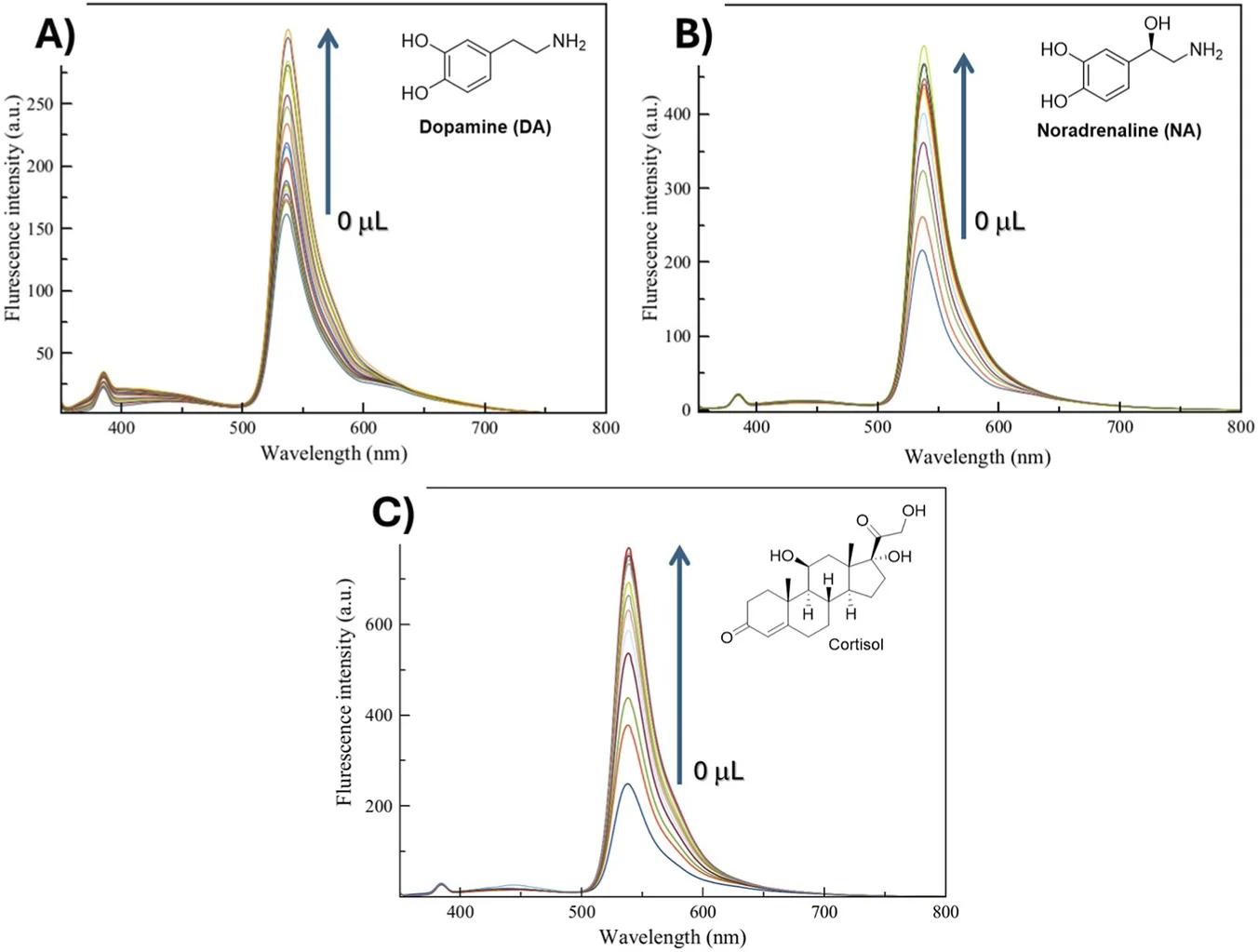
**(A)** Fluorescence titration spectra of *CNPs-Ar-BDPy* with DA; **(B)** Fluorescence titration spectra of *CNPs-Ar-BDPy* with Nadr; **(C)** Fluorescence titration spectra of *CNPs-Ar-BDPy* with Cortisol in MilliQ water pH = 7.

The sensing properties of the nanosensor were tested in milliQ water (pH = 7). Fluorescence titration was performed by adding increasing aliquots (from 2 to 100 μL) of a 10^-4^ M DA, NAdr and cortisol solution in water to a 0.01 mg mL^-1^ *CNPs-Ar-BDPy* solution. Calibration range (from 1 × 10^−7^ to 1 × 10^−5^ M). With the progressive additions of the analytes, the *CNPs-Ar-BDPy* showed a progressive increase in fluorescence intensity (Figures 3A-C respectively). All measurements are triplicates of three independent measurements.

Table 1 shows the apparent affinity constants (K), obtained by HypSpec software, and the relative limit of detection values (Lim et al., 2026, Legnani et al. 2020). In particular, *CNPs-Ar-BDPy* shows K values, in terms of log, of 6.56, 7.27 and 7.02 for DA, NAdr and cortisol, respectively (see Supplementary Figures S8-S10). Specifically, the calculated detection limit values are in the order of ppb or lower, with a blank standard deviation of 0.019987 for the NAdr titration, 0.019992 for the DA titration, and 0.019989 for the cortisol titration.

**TABLE 1:** Table with the values of apparent affinity constants for the fluorescence titration of CNPsAr-BDPy, BDPy-Ar-OH, CNPs-COOH and CNPs-Ethyl-BDPy with DA, NAdr and cortisol, calculated using λex 370 nm in MilliQ water (pH=7), λex 500 nm in ethanol, λex 370 nm in MilliQ water (pH=7) and λex 370 nm in ethanol, respectively.

Receptor	Solvent	λex (nm)	log K (DA) and LOD	log K (NAdr) and LOD	log K (Cortisol) and LOD	F.B.
**CNPs-Ar-BDPy**	MilliQ water	370	6.56 ± 0.04 1.5 ppb	7.27± 0.03 0.18 ppb	7.02 ± 0.02 0.13 ppb	Turn-on
**BDPy-Ar-OH**	Ethanol	500	4.93 ± 0.04	4.35 ± 0.04	5.19 ± 0.03	Weak turn-off
**CNPs-COOH**	MilliQ water	370	4.31 ± 0.01	4.60 ± 0.01	5.37 ± 0.01	Weak turn-off
**CNPs-Ethyl- BDPy**	Ethanol	370	4.86 ± 0.014	5.51 ± 0.003	5.29 ± 0.006	Turn-off

(K) = apparent affinity constant, LOD = calculated with3s/K, (F.B.) = Fluorescent behavior.

FT-IR measurements were performed to confirm the formation of hydrogen bonds between Dopamine and Bodipy moiety. In particular, comparing the FT-IR spectrum of *CNPs-Ar-BDPy*, in the spectrum of *CNPs-Ar-BDPy*@DA (Supplementary Figure S5) the signal at 1,277 cm^-1^ corresponding to the B-F stretching disappears and the peak at 1,287 cm^-1^ corresponding to the vibrational stretching of the OH catechol groups shift to lower energies (1,295 cm^-1^), supporting that a possible sensing mechanism involves the B-F portion of BDPy bound to the CNPs and the catechol portion of DA. This supports the formation of hydrogen bonds between the fluorines of BDPy, and the catechol portion of DA. Similar behavior has been previously observed in literature, where DFT studies supported the hydrogen bonds between fluorine atoms of a Bodipy receptor, and DA (Puglisi et al., 2024a). Additional studies would be needed to further support the role of B-F···H-O interactions, including complementary mechanistic experiments and/or computational analysis.

To shed light on the sensing mechanism, and in particular to understand the role of the carbon nanoparticle core and the fluorophore into the sensing properties, we performed fluorescence titrations of DA, NAdr and cortisol using Ar-BDPy and CNPs-COOH as receptors (Supplementary Figures S11-S20).

In addition, we synthesized similar Bodipy functionalized carbon nanoparticles (Scheme S1 in the ESI) bearing a non-conjugated linker, *CNPs-Ethyl-BDPy*, to evaluate the importance of maintaining electronic conjugation between the graphitic sheets of the CNPs core and the BDPy recognition site. Apparent affinity constants for these three receptors were also calculated, observing a decrease in the apparent affinity constants values by almost two orders of magnitude, highlighting the role of carbon nanoparticles in the sensing and the importance of the electronic conjugation between recognition site and the core of the nanoparticles. The thermodynamic difference is more than two orders of magnitude, given that water is a more competitive solvent than ethanol, but we cannot quantify it due to solubility issues.

Table 1 shows the apparent affinity constants (K) and fluorescent behavior (F.B), obtained by HypSpec software. In particular, BDPy-Ar-OH shows K constant values, in terms of log, of 4.93, 4.35 and 5.19 for DA, NAdr and cortisol, respectively, CNPs-COOH shows K constant values, in terms of log, of 4.31, 4.60 and 4.37 for DA, NAdr and cortisol, respectively; finally *CNPs-Ethyl-BDPy* K constants were calculated in ethanol (due to the low solubility in water), using an λ_ex_ of 490 nm (see Supplementary Figures S24-S26).

### 3.4 Selectivity

Selectivity is a crucial parameter for a sensor. In particular, the possibility to discriminate against one analyte for the other is essential to avoid false positives responses. In this context, a single sensor can be evaluated in terms of selectivity with respect to three different analytes (DA, NAdr and cortisol) considering the full titration profiles of the nanosensor with the stress biomarkers tested.

**FIGURE 4:**
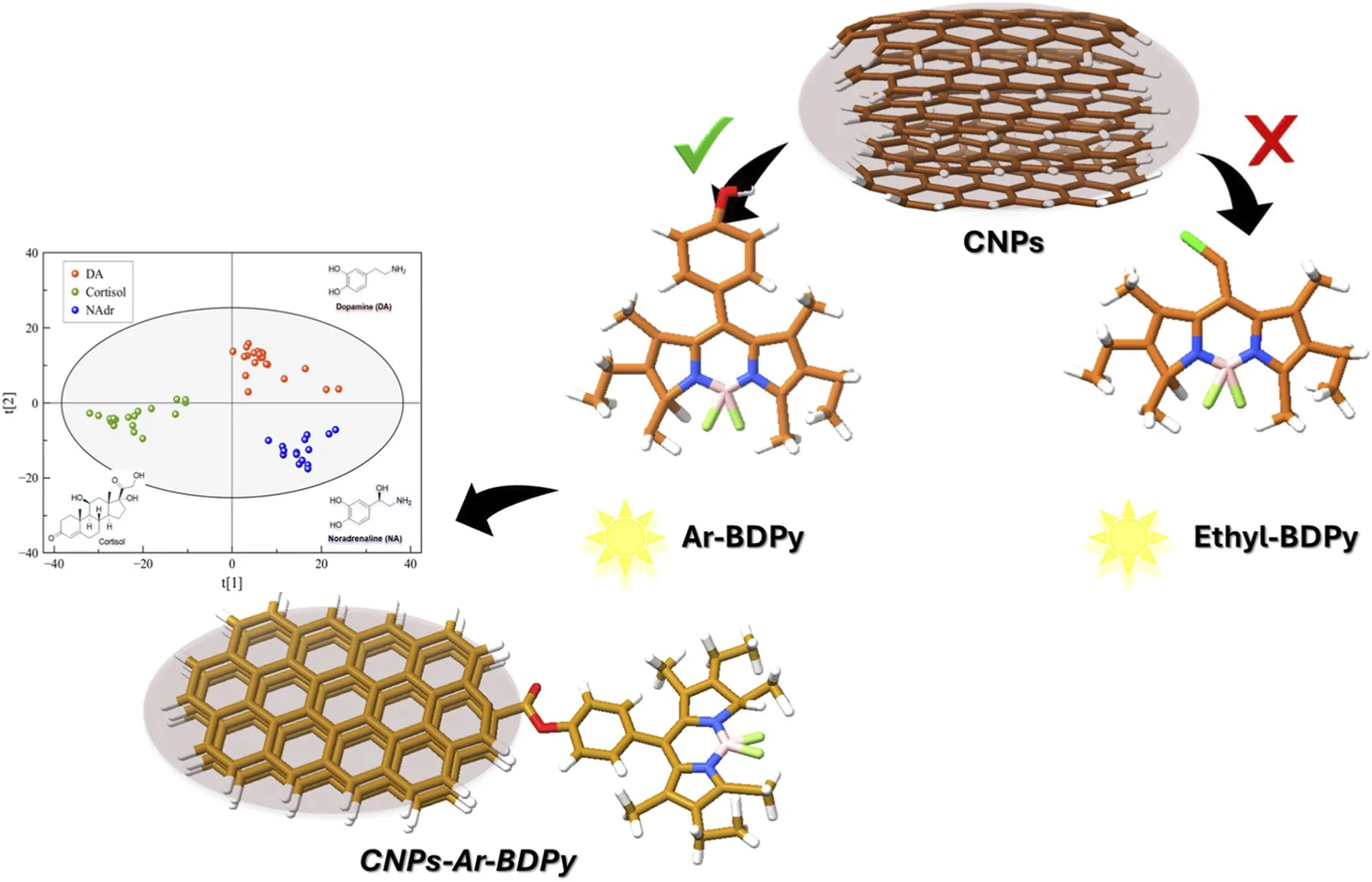
PLS-DA of the full titration between *CNPs-Ar-BDPy* and DA, Nadr and Cortisol.

Multivariate analysis using a Partial Least Squares Discriminant Analysis (PLS-DA) model built by importing all titration spectra of *CNPs-Ar-BDPy* against DA, NAdr, and cortisol into the multivariate analysis software, provided good clustering of the three human biomarkers (see Figure 4; Supplementary Table S3). This selectivity, determined using PLS-DA, is the result of processing all titration data for each individual analyte.

In order to assess the practical potential of the nanosensor in real sample, such as saliva, we added adrenaline, glucose, uric acid, NaNO2, urea, KNO3, and KH2PO4 at 10 μM.

These analytes are commonly present in human saliva (Ngamchuea et al., 2018) (see Supplementary Table S1 in the ESI) and recorded the corresponding fluorescence spectra. The emission intensities of the native *CNPsAr-BDPy* were then compared with those obtained upon titration with DA, NAdr, and cortisol (1 μM each), as discussed above. As shown in Supplementary Figure S27, no substantial changes in the CNPs emission were observed relative to the tested biomarkers.

## 4 Conclusion

In this work, a fluorescent sensor using BDPy-based carbon nanoparticle that can detect and discriminate DA, NAdr, and cortisol in water has been developed. Our nanosensor shows excellent apparent affinity constants affinities towards the biomarkers tested, with high selectivity, as PLS-DA demonstrated. The importance of the nanoparticle core, as well as the covalent aromatic link with the bodipy fluorophore has been provided by control experiments by using single bodipy and other nanoparticles, non-functionalized and alkyl decorated. The FT-IR results support the interaction between dopamine and BODIPY-functionalized nanoparticles; however, as part of future work, computational analyses will be conducted to further support the proposed role of B-F···H-O interactions.

The *CNPs-Ar-BDPy* reported in this work lay the foundations for future developments of the latter in a strip test of solid-state nanoparticles, differently functionalized with different fluorophores, in order to cover a broad UV-Visible spectrum and create a *point-of-care* carbon nanoparticle sensor for realtime monitoring of catecholamine and cortisol levels and, more generally, human health conditions.

Further validation of the method’s practicality and reliability will also require the assessment of emerging analytical figures of merit such as CACI and AMRI (Fuente-Ballesteros et al., 2026; Mansour et al., 2026).

## Data Availability

The original contributions presented in the study are included in the article/Supplementary Material, further inquiries can be directed to the corresponding author.
