# Supplementary material for: Sensing of human stress biomarkers using BODIPY-functionalized carbon nanoparticles
Source: Front Chem. 2026 Aug 25;14:1849599. doi: 10.3389/fchem.2026.1849599 (PMC13550218; doi:10.3389/fchem.2026.1849599)
Supplement: Supplementary file 1 [file DataSheet1.docx]

Sensing of Human Biomarkers Using BODIPY-Functionalized Carbon Nanoparticles

Alessia Cavallaro^1^, Micheal Spinello^1^, Victor Sebastian^2,3,4,5^, Angelo Ferlazzo^1^, Antonino Gulino^1^, Roberta Ruffino^1,6^, Giovanni Li Destri^1,6^, Andrea Pappalardo^1^, Nunzio Tuccitto^1,4^, Giuseppe Trusso Sfrazzetto^1^ and Rossella Santonocito^1^*

Supplementary Material

**Table of contents**

[1 Chemical characterization of *CNPs-Ar-BDPy*	2](#_Toc231567733)

[^1^H NMR	2](#_Toc231567734)

[XPS	3](#_Toc231567735)

[FT-IR	4](#_Toc231567736)

[2 Morphological characterization of CNPs-Ar-BDPy	5](#_Toc231567737)

[3 Optical characterization	6](#_Toc231567738)

[4 HypSpec output for the titration of CNPs-Ar-BDPy	6](#_Toc231567739)

[5 HypSpec output for the titration of BDPy-Ar	8](#_Toc231567740)

[6 HypSpec output for the titration of CNPs-COOH	11](#_Toc231567741)

[7 Synthesis and Chemical characterization of CNPs-Ethyl-BDPy	14](#_Toc231567742)

[8 Selectivity test: multivariate Analysis	18](#_Toc231567743)

[9 Stability test	19](#_Toc231567744)

**Table S1.** Standard values for catecholamines and cortisol

|  | Plasma/Blood | Urine(24h) | Saliva |
| --- | --- | --- | --- |
| **Adrenaline** | 0-140 pg/ml | 15-80 μg/24h | 10 pg/ml |
| **Noradrenaline** | 70-1700 pg/ml | 0.5-20 μg/24h | 10-20 pg/ml |
| **Dopamine** | 0-30 pg/ml | 65-400 μg/24h | 5-50 pg/ml |
| **Cortisol** | 9-24 μg/dl (morning)  3-12 μg/dl (evening) | 20/100 μg/dl | 3-10 ng/ml (morning)  0.6-2.50 ng/ml (evening) |

# 1 Chemical characterization of *CNPs-Ar-BDPy*

# ^1^H NMR

**Supplementary Figure S1.** ^1^H NMR spectrum of CNPs-Ar-BDPy in CDCl_3_

Supplementary Figure 2

## XPS

Supplementary Table S1. XPS Binding Energies and assignments.

| **Atomic state** | **B. E. (eV)** | **Assignment** |
| --- | --- | --- |
| **C 1s** | 284.7  285.0  286.0  286.9  288.1 | sp^2^  sp^3^ C-C, C-H  C-N  C-O  -COO |
| **O 1s** | 531.6  532.7 | -C=O  -C-O-BDPy |
|  | 534.1 | phenolic |
| **N 1s** | 399.3  400.2 | pyrrole ring  quaternized pyrrole |
| **F 1s** | 685.9 | BDPy |
|  | 687.7 | pentafluorophenol |

## FT-IR

Supplementary Figure S3. Comparison of the FT-IR spectra of CNPs-5FPh with CNPs-Ar-BDPy

**Supplementary Figure S4.** FT-IR spectrum of Dopamine (DA)

The DA spectrum shows the diagnostic signals, including the stretching vibration peak of the catechol structure at a wavenumber of 1287 cm^-1^, the stretching vibration peak of the CQC bond in the dopamine benzene ring at around 1615 cm^-1^, and the N–H bond stretching vibration at 3157 cm^-1^ and 3332 cm^-1^.

Supplementary Figure S5. Comparison of the FT-IR spectra of *CNPs-Ar-BDPy* (in red) with CNPs-Ar-BDPy@DA (in black).

# 2 Morphological characterization of CNPs-Ar-BDPy

Supplementary Figure S6. AFM

# 3 Optical characterization

Supplementary Figure S7. Optical characterization of CNPs-Ar-BDPy: a) UV-Visible spectrum (0.1 mg mL^-1^ in MilliQ water pH=7) and b) fluorescence spectra in the excitation range 280-460 nm (0.1 mg mL^-1^ in MilliQ water pH=7).

# 4 HypSpec output for the titration of CNPs-Ar-BDPy

Supplementary Figure S8. HypSpec output file for fluorescence titration of CNPs-Ar-BDPy with DA

Project title:

Converged in 5 iterations with sigma = 6,3457

standard

Log beta	value	deviation

AB	**6.5643**	0.0046

Supplementary Figure S9. HypSpec output file for fluorescence titration of CNPs-Ar-BDPy with NAdr

Project title:

Converged in 1 iteration with sigma = 5,3941

standard

Log beta	value	deviation

AB	7.2668	0.0042

**Supplementary Figure S10**. HypSpec output file for fluorescence titration of CNPs-Ar-BDPy with cortisol

Project title:

Converged in 4 iterations with sigma = 3,1324

standard

Log beta	value	deviation

AB	7.0210	0.004

# 5 HypSpec output for the titration of BDPy-Ar

**Supplementary Figure S11.** Fluorescence titration spectra of BDPy-Ar with DA, Nadr and Cortisol in ethanol.

Supplementary Figure S12. HypSpec output file for fluorescence titration of BDPy-Ar with DA

Project title:

Converged in 4 iterations with sigma = 3,1324

standard

Log beta	value	deviation

AB	4.9376	0.004

Supplementary Figure S13. HypSpec output file for fluorescence titration of BDPy-Ar with NAdr

Project title:

Converged in 3 iterations with sigma = 3,7213

standard

Log beta	value	deviation

AB	4.3537	0.0454

Supplementary Figure S14. HypSpec output file for fluorescence titration of BDPy-Ar with Cortisol

Project title:

Converged in 4 iterations with sigma = 2,9669

standard

Log beta	value	deviation

AB	5.1966	0.0035

# 6 HypSpec output for the titration of CNPs-COOH

Supplementary Figure S15. Fluorescence titration spectra of CNPs-COOH with DA in milliQ water (pH=7).

Supplementary Figure S16. Fluorescence titration spectra of CNPs-COOH with DA in milliQ water (pH=7).

Project title:

Converged in 3 iterations with sigma = 6,3842

standard

Log beta	value	deviation

AB	4.3134	0.0149

Supplementary Figure S17. Fluorescence titration spectra of CNPs-COOH with NAdr in milliQ water (pH=7).

Supplementary Figure S18. Fluorescence titration spectra of CNPs-COOH with NAdr in milliQ water (pH=7).

Project title:

Converged in 4 iterations with sigma = 2,6596

standard

Log beta	value	deviation

AB	4.6017	0.0034

Supplementary Figure S19. Fluorescence titration spectra of CNPs-COOH with cortisol in milliQ water (pH=7).

Supplementary Figure S20. Fluorescence titration spectra of CNPs-COOH with cortisol in milliQ water (pH=7).

Project title:

Converged in 3 iterations with sigma = 3,8142

standard

Log beta	value	deviation

AB	4.3785	0.0068

# 7 Synthesis and Chemical characterization of CNPs-Ethyl-BDPy

^1^H NMR

**Supplementary Scheme S1.** Schematic pathway showing the reactions to obtain CNPs-Ethyl-BDPy: (a) ethanolamine, 130°C, (b) DIPEA, DMSO, RT, N_2_, 3 days.

Supplementary Figure S21. ^1^H NMR spectrum of CNPs-CONH(CH_2_)_2_OH in D_2_O.

**Supplementary Figure S22.** ^1^H NMR spectrum of CNPs-Ethyl-BDPy in CDCl_3_.

Supplementary Figure S23. a) Comparison of UV-Vis spectra of CNPs-BDpy-CH_2_ in water (black), CNPs-BDPy-CH_2_ in EtOH (red) and BDPy-CH_2_-Cl in EtOH (blue); b) Comparison of emission spectra of CNPs-BDpy-CH_2_ in water (black), CNPs-BDPy-CH_2_ in EtOH (red) and BDPy-CH_2_-Cl in EtOH (blue) using l ex= 370 nm; c) Comparison of emission spectra of CNPs-BDpy-CH_2_ in water (black), CNPs-BDPy-CH_2_ in EtOH (red) and BDPy-CH_2_-Cl in EtOH (blue) using l ex= 490 nm

Supplementary Figure S24. Fluorescence titration spectra of CNPs-BDPy-CH_2_ with DA, in ethanol.

Supplementary Figure S25. Fluorescence titration spectra of CNPs-BDPy-CH_2_ with NAdr, in ethanol.

Supplementary Figure S26. Fluorescence titration spectra of CNPs-BDPy-CH_2_ with Cortisol, in ethanol.

# 8  Selectivity test: multivariate Analysis

|  | **Members** | **Correct** | **DA** | **Cortisol** | **Nadr** | **No class (YPred <= 0)** |
| --- | --- | --- | --- | --- | --- | --- |
| **DA** | 22 | 95,45% | 21 | 0 | 1 | 0 |
| **Cortisol** | 18 | 100% | 0 | 18 | 0 | 0 |
| **Nadr** | 15 | 100% | 0 | 0 | 15 | 0 |
| **No class** | 0 |  | 0 | 0 | 0 | 0 |
| Total | 55 | 98,18% | 21 | 18 | 16 | 0 |
| Fisher's prob. | 1,2e-023 |  |  |  |  |  |

Supplementary Table S3. Misclassification Table

**Supplementary Figure S27.** A) Fluorescence spectra of CNPs-Ar-BDPy, titration with DA, NAdr, and cortisol at the concentration of 1μM, and the addition of 10 μM of commonly reported interferents in saliva. B) Histogram showing the fluorescence intensity values at 536 nm for the biomarkers and interferents; the error bars represent the standard deviation of three different replicates.

# 9 Stability test

We verified the stability of the CNPs-Ar-BDPy nanosensor by recording its fluorescence spectrum at intervals of several days, and after one week, we found that the nanoparticles’ fluorescence emission remained stable; furthermore, we observed no presence of precipitates. The stability inferred from the fluorescence spectrum refers to optical stability under the tested conditions and should not be interpreted as a full colloidal stability assessment.

**Supplementary Figure S28.** Fluorescence spectra of the CNPs-Ar-BDPy nanosensor recorded over several days in milliQ water (pH=7) at a concentration of 0.01 mg/mL using λex=370 nm.

Supplementary Figure S29. HypSpec output file for fluorescence titration of [CNPs-Ar-BDPy]= 1 x 10^-6^ M with DA

Project title:

Converged in 1 iterations with sigma = 13,370

standard

Log beta	value	deviation

AB	7.037	0.0251

Supplementary Figure S31. HypSpec output file for fluorescence titration of [CNPs-Ar-BDPy]= 1 x 10^-8^ M with DA

Project title:

Converged in 1 iterations with sigma = 7,7064

standard

Log beta	value	deviation

AB	6.6929	0.0055
